# Cylinder Bicuspid Pulmonary Valve Reconstruction Using Equine Pericardium: A Novel Technique for Right Ventricular Outflow Tract Repair

**DOI:** 10.3390/jcm15041457

**Published:** 2026-02-12

**Authors:** Ahmed F. Elmahrouk, Abdelmonem M. Helal, Mohammad F. Babgi, Abdulbadee Bogis, Amjad A. Kouatli, Mohammad S. Shihata

**Affiliations:** 1 Division of Cardiac Surgery, Cardiovascular Department, King Faisal Specialist Hospital and Research Center, Jeddah 21499, Saudi Arabia; mshihata@gmail.com; 2 Cardiothoracic Surgery Department, Tanta University, Tanta 31527, Egypt; 3 Pediatric Cardiology Department, King Faisal Specialist Hospital and Research Center, Jeddah 23433, Saudi Arabia; habdelmonem@kfshrc.edu.sa (A.M.H.); akouatli@kfshrc.edu.sa (A.A.K.); 4 Pediatric Cardiology Division, Department of Pediatrics, Cairo University, Cairo 12613, Egypt; 5 Department of Cardiac Surgery, Cardiac Center, King Abdullah Medical City, Makkah 24246, Saudi Arabia; mfbabgi@gmail.com; 6 Department of Surgery, Cardiac Surgery Section, King Abdulaziz University, Jeddah 21589, Saudi Arabia; dr.abdulbadee@gmail.com

**Keywords:** pulmonary hypoplasia, pulmonary atresia, right ventricular outflow tract obstruction

## Abstract

**Background**: Right ventricular outflow tract (RVOT) reconstruction is frequently performed for pediatric patients with pulmonary valve anomalies, yet optimal techniques remain debated. The equine pericardium offers a promising substrate for pulmonary valve reconstruction but has been understudied in pulmonary valve reconstruction. This study evaluated a novel technique using the equine pericardium to create a cylinder bicuspid pulmonary valve for RVOT reconstruction. **Methods**: In this retrospective cohort study, 17 pediatric patients (median age: 10 months; 53% male) underwent RVOT reconstruction with equine pericardium between 2023 and 2024. The valve was fashioned from a patch of equine pericardium into a cylinder to create a functionally bicuspid valve. The height of the cylinder ranged from 1.5–2 cm. The diameter was measured around a Hegar dilator corresponding to a valve size z-score of +3. The outcomes included the degree of postoperative pulmonary regurgitation, RVOT pressure gradients, postoperative complications, and reinterventions. **Results**: Postoperatively, the median peak RVOT pressure gradient decreased significantly from 70 mmHg (IQR: 65–90) to 25 mmHg (IQR: 20–40; *p* < 0.001). Mild pulmonary regurgitation persisted in one patient (5.9%). Five patients had mild right ventricular dysfunction (29.41%). At a median 3-month follow-up (IQR: 1–8), 17.7% (*n* = 3) underwent cardiac catheterization. There was no postoperative mortality. **Conclusions**: Cylinder bicuspid pulmonary valve reconstruction using the equine pericardium effectively reduces RVOT obstruction while maintaining pulmonary valve competence and demonstrates acceptable short-term safety. Having a competent pulmonary valve after repairing the hypoplastic pulmonary valve annulus is very promising; however, the small cohort and limited follow-up preclude definitive conclusions about long-term durability. Larger prospective studies with longer follow-up periods are needed to validate this technique for RVOT reconstruction.

## 1. Background

Right ventricular outflow tract (RVOT) reconstruction is one of the most common and critical surgical interventions for pediatric patients with congenital heart disease (CHD), addressing a spectrum of anomalies ranging from pulmonary stenosis to atresia [1]. The primary objectives of this procedure are to restore physiological blood flow from the right ventricle to the pulmonary arteries, alleviate chronic right ventricular strain, and mitigate long-term risks of pulmonary valve insufficiency, ventricular dysfunction, and heart failure [2,3]. Despite its clinical importance, the optimal surgical approach remains debated, with current techniques encompassing valveless repairs, synthetic conduits, xenografts (e.g., bovine/porcine pericardial or aortic tissues), homografts, and autologous pericardial valved conduits [4]. Each method involves trade-offs: valveless repairs, while avoiding prosthesis-related complications, may exacerbate regurgitation, whereas prosthetic or biological conduits face challenges such as limited growth potential, structural degeneration, high cost, especially in developing countries, and calcification—particularly problematic in growing pediatric patients [4,5,6,7].

The quest for an ideal RVOT reconstruction material has led to a growing interest in tissue-engineered and decellularized biomaterials. Decellularization is a process that removes the cellular components from a tissue, leaving behind an acellular scaffold that is less likely to elicit an immune response and may have the potential for remodeling and regeneration [8]. Renewed interest in the equine pericardium has emerged because of its favorable biocompatibility, pliability for precise surgical shaping, and potential resistance to calcification compared with other xenografts [8]. While the use of equine pericardium in various congenital cardiac repairs has been reported with encouraging results, its application for creating a functional, patient-specific pulmonary valve remains a novel and understudied area [8,9].

This study introduces a novel technique for RVOT reconstruction using a cylinder bicuspid pulmonary valve constructed from decellularized equine pericardium. The aim of this study is to evaluate the early and mid-term outcomes of this technique in a cohort of pediatric patients, with a focus on its effectiveness in relieving RVOT obstruction while maintaining pulmonary valve competence. We hypothesize that this novel approach will provide a durable solution for RVOT reconstruction, potentially reducing the need for reinterventions and improving long-term outcomes for this challenging patient population.

## 2. Methods

### 2.1. Study Design and Patient Cohort

This retrospective cohort study was conducted at King Faisal Specialized Hospital and Research Center, Jeddah, Saudi Arabia, and included 17 consecutive pediatric patients who underwent right ventricular outflow tract (RVOT) reconstruction using a novel cylinder bicuspid pulmonary valve constructed from equine pericardium between May 2023 and October 2024. The study was approved by the Institutional Review Board (IRB) of King Faisal Specialist Hospital & Research Center (Reference-RAC-2251125), which waived the need for individual patient consent due to the retrospective nature of the analysis. All procedures were performed in accordance with the ethical standards of the institutional and/or national research committee and the Helsinki declaration.

Inclusion criteria comprised pediatric patients (age < 18 years) with congenital heart defects requiring surgical intervention for severe RVOT obstruction, defined as a peak pressure gradient > 50 mmHg, who required a transannular patch for pulmonary valve repair. This included patients with pulmonary valve hypoplasia or atresia.

Exclusion criteria were patients with mild RVOT obstruction not requiring a transannular patch, those who underwent a different type of valve reconstruction (e.g., homograft, bovine jugular vein conduit), and patients with complicating factors such as Atrioventricular canal, Major Aorto-Pulmonary Collateral Arteries (MAPCAs). We also clarify that patients with active endocarditis, severe distal pulmonary artery hypoplasia precluding standardized reconstruction, or prior failed RVOT conduit within 3 months were excluded, as well as patients with active infection at the time of surgery. Patients with incomplete medical records were also excluded from the analysis.

### 2.2. Technique

**Figure 1:**
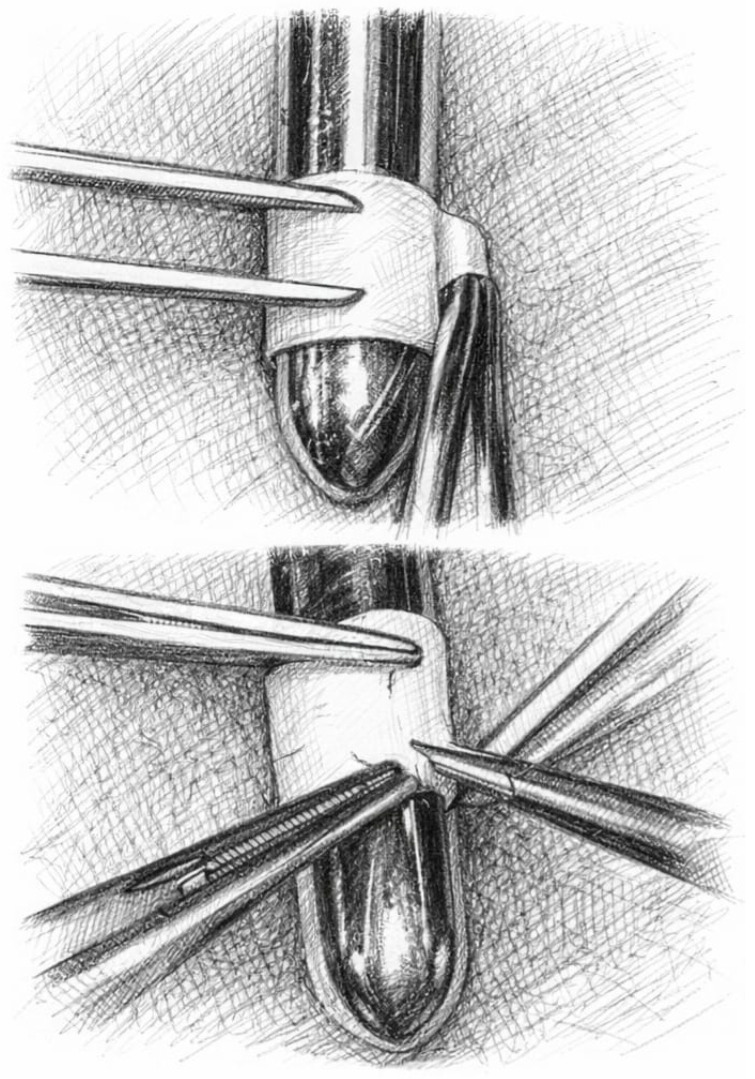
The cylinder diameter was determined by wrapping the pericardial patch around a Hegar dilator corresponding to a valve size Z-score of +3 for the patient’s body surface area (BSA).

The novel cylinder bicuspid pulmonary valve was constructed from a decellularized equine pericardial patch (Matrix P^®^; AutoTissue GmbH, Berlin, Germany). The patch was tailored to create a cylinder with a height ranging from 1.5 to 2.0 cm, depending on the patient’s anatomy. The cylinder diameter was determined by wrapping the pericardial patch around a Hegar dilator corresponding to a valve size Z-score of +3 for the patient’s body surface area (BSA) (Figure 1). This oversizing strategy was adopted to accommodate future somatic growth potential in pediatric patients and to minimize the risk of late stenosis. We have also avoided excessive oversizing that could predispose to significant pulmonary regurgitation.

The cylinder itself acts as the bileaflet valve once fixed in position and is inspired by stentless aortic valve tissue prostheses. However, the fact that it is made of a single layer of equine pericardial patch makes it easier to implant and more versatile in accommodating different RVOT and pulmonary artery geometries in patients with TOF.

**Figure 2:**
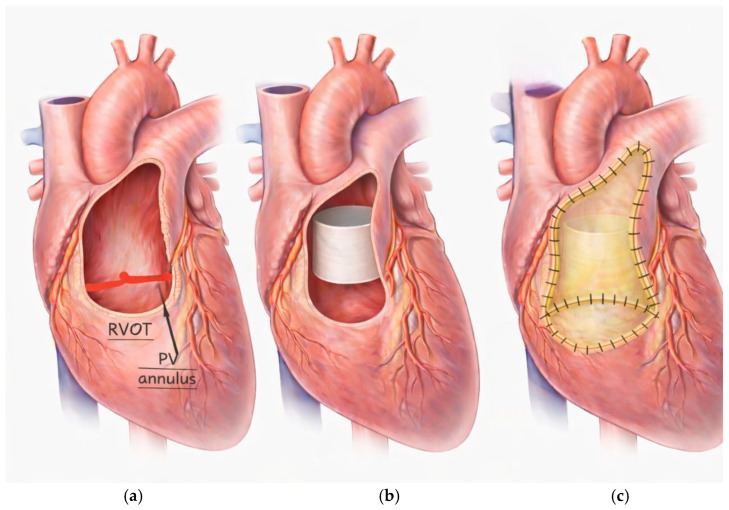
(**a**) The main pulmonary artery was opened, and the native pulmonary valve was excised. (**b**) The equine pericardial cylinder is fixed in position by four anchoring sutures extending from the native pulmonary valve annulus to just below the pulmonary artery bifurcation. (**c**) A separate patch is used for augmentation of the proximal left and main pulmonary arteries.

All surgeries were performed via a median sternotomy with the patient under general anesthesia. Cardiopulmonary bypass (CPB) was established with aortic and bicaval cannulation. Myocardial protection was achieved with antegrade cold blood cardioplegia. After initiating CPB, the main pulmonary artery was opened, and the native pulmonary valve was excised. The RVOT was then prepared, and any obstructing muscle bundles were resected (Figure 2).

**Figure 3:**
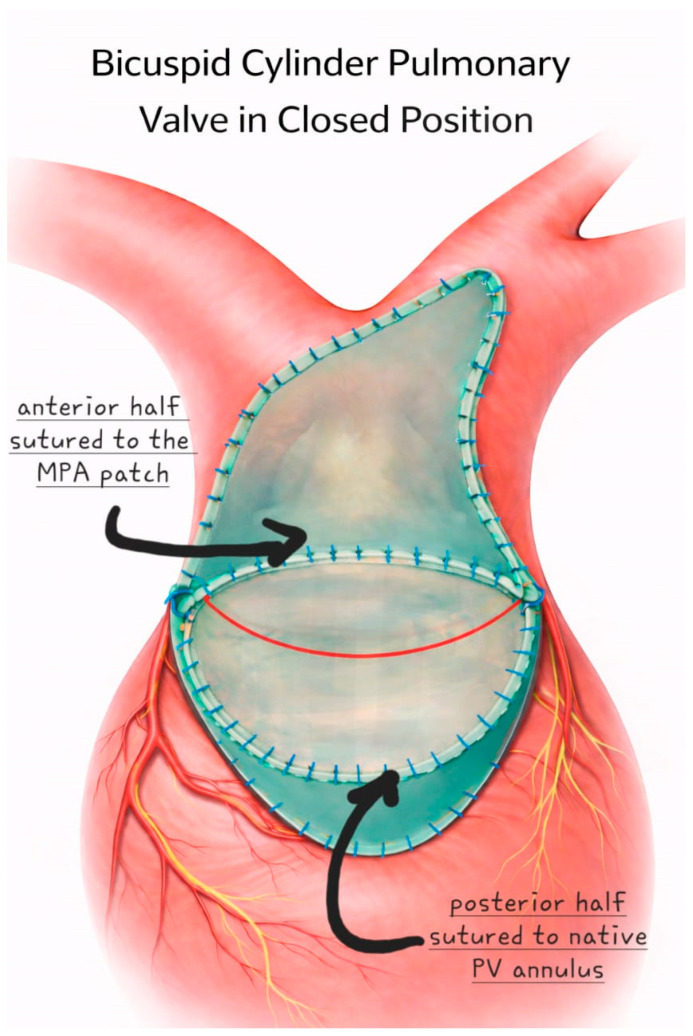
A separate patch is used for augmentation of the proximal left and main pulmonary arteries. The sides of the cylinder are incorporated in the augmentation patch suture line. The red line represents the coaptation line of the bicuspid valve.

The equine pericardial cylinder is fixed in position by four anchoring sutures extending from the native pulmonary valve annulus to just below the pulmonary artery bifurcation. A separate patch is used for augmentation of the proximal left and main pulmonary arteries. The sides of the cylinder are incorporated in the augmentation patch suture line. Proximally, the posterior half is sewn to the native pulmonary valve annulus. The MPA patch is folded at the level of the new cylinder valve annulus and sewn to the anterior half of the cylinder valve. The remainder of the patch is then used to augment the RVOT below the valve. The distal end of the cylinder is left free to act as a functionally bicuspid valve (Figure 3). The surgical technique is illustrated in Video S1.

Intraoperative transesophageal echocardiography (TEE) was performed in all cases to confirm proper valve function, assess leaflet mobility and coaptation, and rule out any paravalvular leaks before weaning the patient from CPB.

### 2.3. Data and Outcomes

Data were retrospectively collected from the hospital’s electronic health records and surgical database. Preoperative data included patient demographics (age, sex, height, weight, BSA), primary cardiac diagnosis, associated congenital anomalies, and preoperative echocardiographic measurements, including the peak RVOT pressure gradient and pulmonary valve annulus diameter. Operative data included the type of surgery, CPB time, and aortic cross-clamp (ischemic) time.

The primary outcomes of the study were the postoperative peak RVOT pressure gradient and the degree of pulmonary regurgitation (PR) at the time of discharge and at the latest follow-up. PR was graded as none, trivial, mild, moderate, or severe based on color Doppler jet width and the pressure half-time of the regurgitant jet on transthoracic echocardiography.

Secondary outcomes included postoperative complications such as the need for extracorporeal membrane oxygenation (ECMO), incidence of complete heart block (CHB), right and left ventricular dysfunction, need for anti-failure medications, duration of intensive care unit (ICU) and hospital stay, and in-hospital mortality. Follow-up data were collected from outpatient clinic visits and included information on reinterventions (surgical or catheter-based) and survival.

### 2.4. Statistical Analysis

The data were analyzed via descriptive and inferential statistics. Continuous variables with a normal distribution are expressed as the mean ± standard deviation (SD), whereas nonnormally distributed variables are reported as the median (interquartile range, IQR). Categorical variables are summarized as frequencies and percentages.

The Wilcoxon signed-rank test was employed to compare pre- and postoperative right ventricular outflow tract (RVOT) pressure gradients (PGs), given the nonparametric distribution of the data. A two-tailed *p* value < 0.05 was considered statistically significant. All analyses were performed via STATA BE 18 (Stata Corp., College Station, TX, USA).

## 3. Results

### 3.1. Preoperative and Operative Data

The study cohort consisted of 17 pediatric patients, with a slight male predominance (*n* = 9, 52.9%). The median age at the time of surgery was 10 months (IQR: 7–11 months), and the median weight was 8.01 kg (IQR: 7.0–9.0 kg). Down syndrome was present in three patients (18.75%). The primary indication for surgery was severe RVOT obstruction, with a median preoperative peak pressure gradient of 70 mmHg (IQR: 65–90 mmHg). The RVOT obstruction was multi-level in the majority of patients, with valvular obstruction being the most common (94.1%), followed by subvalvular (64.7%) and supravalvular (35.3%) obstruction. One patient (5.9%) had pulmonary atresia. The mean preoperative pulmonary valve annulus diameter was 4.73 ± 1.10 mm, corresponding to a Z-score of −2.5 ± 0.8. Associated cardiac anomalies were present in 64.7% of patients, with patent ductus arteriosus (PDA) being the most common (41.2%). The detailed baseline characteristics and preoperative data are presented in Table 1.

### 3.2. Postoperative Data

All 17 patients successfully underwent RVOT reconstruction with the novel cylinder bicuspid pulmonary valve. The median CPB time was 93 min (IQR: 66–101 min), and the median aortic cross-clamp time was 58 min (IQR: 45–72 min). Associated procedures were performed in 58.8% of patients, with PDA ligation being the most common (35.3%).

**Figure 4:**
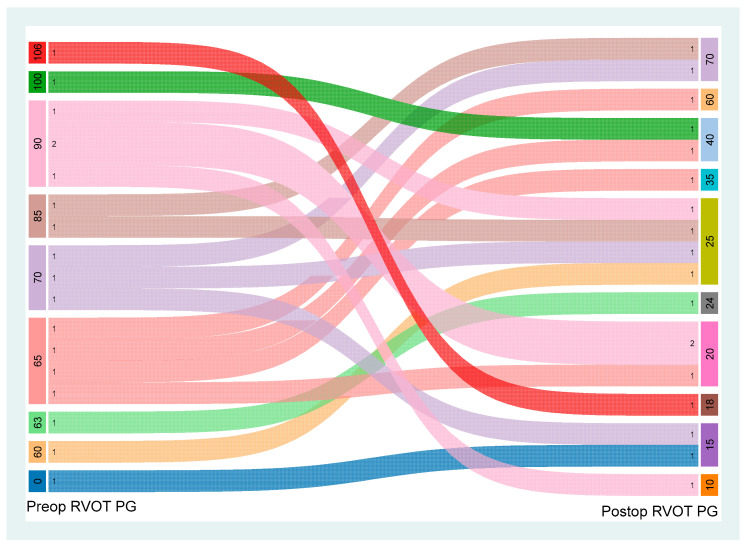
Alluvial graph of changes in the right ventricular outflow tract (RVOT) peak pressure gradient (PG) (mmHg) preoperatively and postoperatively. A preoperative RVOT PG of zero indicates pulmonary atresia.

There was a statistically significant reduction in the peak RVOT pressure gradient from a median of 70 mmHg preoperatively to 25 mmHg postoperatively (*p* < 0.001), as illustrated in Figure 4. At the time of discharge, only one patient (5.9%) had mild pulmonary regurgitation, while the remaining 16 patients (94.1%) had no or trivial PR.

There were no in-hospital mortalities. Postoperative complications were infrequent. Five patients (29.4%) had mild right ventricular dysfunction, and one patient (5.9%) had moderate right ventricular dysfunction, which improved with medical management. Three patients (17.6%) had mild left ventricular dysfunction. No patient required ECMO support or developed complete heart block. The median ICU stay was 8 days (IQR: 6–9 days), and the median total hospital stay was 11 days (IQR: 8–15 days). The detailed postoperative outcomes are summarized in Table 2.

### 3.3. Follow-Up

The median follow-up duration was 3 months (IQR: 1–8 months). During the follow-up period, there were no mortalities. Three patients (17.6%) required catheter-based reintervention. Two patients developed recurrent pulmonary stenosis and underwent successful balloon valvuloplasty, with a significant reduction in the pressure gradient and preservation of valve competence. One patient underwent device closure of a small residual ventricular septal defect (VSD). At the latest follow-up, all patients were in New York Heart Association (NYHA) class I or II.

## 4. Discussion

This study assessed the outcomes of 17 pediatric patients who underwent cylinder bicuspid pulmonary valve reconstruction using equine pericardium. The patient cohort predominantly presented with valvular RVOT obstruction. Surgical intervention significantly reduced the postoperative RVOT peak pressure gradient from a median of 70 mmHg to 25 mmHg. No hospital mortality was reported. Two patients required balloon dilatation for recurrent pulmonary valve stenosis, which could be attributed to undersizing the valve during implantation.

The autologous pericardium does not provide sufficient material for reoperation or staged procedures in patients with congenital heart disease. Therefore, several patch materials have been employed for RVOT repair; however, the optimal material and technique have not yet been identified [6,10]. The ideal patch possesses several key characteristics: it is readily available, easy to handle, resistant to infection, facilitates proper hemostasis, and has low thrombogenicity [11]. Synthetic materials lack growth potential and are prone to infections [12]. Compared with synthetic conduits, biological materials provide better handling and resistance to infection; however, late calcification is a potential limitation in addition to the risk of infection in xenografts [13,14,15]. Tissue-engineered patches were developed to address some of the limitations associated with traditional materials. For example, treated bovine pericardial patches have been engineered to eliminate calcium-binding sites, while dye-mediated photooxidation has been introduced as a replacement for glutaraldehyde fixation [16,17]. RVOT reconstruction using decellularized equine pericardium has attracted interest recently [8]. The equine pericardium showed no structural deterioration in a sheep model and exhibited positive remodeling and regeneration [18]. Weixler and associates [19] evaluated the midterm results of the equine pericardium in 201 patients with several congenital cardiac lesions. They demonstrated good handling and hemostasis; furthermore, the rate of freedom from patch-related reoperation or catheter-based reinterventions was 85% at 2 years [19,20]. In our series, there was no reoperation related to the pulmonary valve, but two catheter-based reinterventions were performed for pulmonary stenosis. We think the reason for post operative gradient in 2 cases, first, we have tried to preserve the integrity of the RV and limit resection through the annulus. Another factor was technically related, especially in our early experience; we probably did not oversize the cylinder enough. Later in our experience, we tend to tie sutures during the equine cylinder construction over an appropriately sized Hegar dilator to avoid purse stringing. This may have minimized the short-term gradient in the rest of the patients.

Survival at 5 years after RVOT reconstruction using homografts was 89%, and freedom from reoperation was 78% at 5 years and 35% at 10 years [21]. Boshnakov and associates reported 85% freedom from reoperation with bovine jugular vein conduits for RVOT reconstruction in truncus arteriosus patients after 5 years, with no difference between bovine jugular vein conduits and homografts [22,23]. On the other hand, fan-shaped Polytetrafluoroethylene (PTFE) valves and valved conduits were associated with 92.3% freedom from reoperation at 10 years, and 79.6% of patients had no or mild pulmonary insufficiency [6]. PTFE mono-cusp reconstruction provides competent and non-stenotic pathways with advantages in terms of midterm remodeling [4]. Only one patient had a mild PR in our series, indicating a competent repair comparable to other techniques.

This study showed that the technique of using equine pericardium for pulmonary valve reconstruction has yielded promising results, particularly in reducing RVOTO and maintaining valve function. This approach may provide a viable alternative for patients with congenital heart defects, potentially improving surgical outcomes and reducing the need for more invasive procedures. These findings suggest that tailored valve reconstruction can enhance patient management strategies and contribute to better long-term cardiac function.

### 4.1. Future Research Directions and Potential Applications

The promising early results of this study warrant further investigation. Future research should focus on several key areas. First, larger, prospective, multicenter studies are needed to validate our findings and to compare the long-term outcomes of this novel technique with those of established RVOT reconstruction methods, such as the use of homografts or bovine jugular vein conduits. These studies should include a comprehensive assessment of valve function, right ventricular remodeling, and the incidence of reintervention over a longer follow-up period.

Second, further research is needed to optimize the surgical technique and to understand the mechanisms of valve failure better. This could include the use of advanced imaging techniques, such as 4D flow MRI, to assess the hemodynamic performance of the reconstructed valve, as well as histological analysis of explanted valves to evaluate the extent of cellular infiltration and tissue remodeling. This information could help to refine the surgical technique and to identify patients who are at higher risk of developing complications.

Third, the potential applications of this technique could be expanded to other areas of congenital heart surgery. For example, the use of custom-built valves from decellularized equine pericardium could be explored for the reconstruction of other cardiac valves, such as the aortic or mitral valve, in pediatric patients and for managing anomalies such as Shone’s complex and hypoplastic left ventricle [24,25]. Furthermore, the principles of this technique could be applied to the development of tissue-engineered heart valves, which have the potential to grow, repair, and remodel, thus providing a truly permanent solution for valvular heart disease.

### 4.2. Limitations

This study has several limitations that should be acknowledged. First, it is a single-center, retrospective study with a small sample size, which limits the generalizability of our findings. Second, the follow-up duration is short, and a longer-term follow-up is needed to preclude robust conclusions about valve durability and long-term performance of the reconstructed valve. A long-term follow-up will also be necessary to address potential long-term issues, including leaflet calcification, structural valve deterioration, progressive pulmonary regurgitation or stenosis. Third, the lack of a control group makes it difficult to draw definitive conclusions about the superiority of this technique over other established methods. Finally, the reinterventions for pulmonary stenosis, although successfully managed with balloon valvuloplasty, highlight the need for further refinement of the surgical technique and sizing of the valve.

## 5. Conclusions

Cylinder bicuspid pulmonary valve reconstruction using the equine pericardium is a safe and effective technique for managing RVOT obstruction in pediatric patients. While the results are encouraging, further research with larger sample sizes and longer follow-up periods is necessary to validate these findings and establish the long-term efficacy and safety of this novel surgical approach.

## Figures and Tables

**Table 1:** Preoperative and operative data of patients who underwent pulmonary valve reconstruction using equine pericardium.

	(*n* = 17)
**Male**	9 (52.94%)
**Age (months)**	10 (7–11)
**Height (cm)**	70.44 ± 8.53
**Weight (kg)**	8.01 ± 1.35
**Body surface area (m^2^)**	0.40 ± 0.06
**Down syndrome**	3 (18.75%)
**Level of RVOT obstruction**	
Subvalvular	11 (64.71%)
Vavular	16 (94.12%)
Supravalvular	6 (35.29%)
Peripheral pulmonary artery stenosis	7 (41.18%)
Pulmonary atresia	1 (5.88%)
**Associate anomaly**	
None	6 (35.29%)
PDA	7 (41.18%)
Others	3 (17.65%)
Muscular VSD	1 (5.88%)
**RVOT pressure gradient (mmHg)**	70 (65–90)
**Preoperative pulmonary valve annulus**	4.73 ± 1.10
**CPB time (min)**	93 (66–101)
**Ischemic time (min)**	58 (45–72)
**Associated surgery**	
None	7 (41.18%)
PDA ligation	6 (35.29%)
PDA ligation + plication of pulmonary branches	1 (5.88%)
Tricuspid valve repair	1 (5.88%)
AVSD repair	1 (5.88%)
PAPVR	1 (5.88%)

Abbreviations: RVOT = right ventricular outflow tract; PAPVR = partial anomaly of pulmonary venous return; AVSD = atrioventricular septal defect; VSD = ventricular septal defect; CPB = cardiopulmonary bypass; PDA = patent ductus arteriosus. Data are presented as mean (SD), median (IQR) or frequency (%).

**Table 2:** Postoperative hospital outcomes of patients who underwent pulmonary valve reconstruction.

	(*n* = 17)
**Hospital mortality**	0
**Diaphragmatic plication**	0
**Complete heart block**	0
**ICU stay** (days)	8 (6–9)
**Postoperative RVOT pressure gradient (mmHg)**	25 (20–40)
**Postoperative mild pulmonary regurgitation**	1 (5.88%)
**Residual VSD**	
Tiny	6 (35.29%)
Moderate	1 (5.88%)
**Right ventricular function**	
Normal	11 (64.71%)
Mild depression	5 (29.41%)
Moderate depression	1 (5.88%)
**Left ventricular dysfunction**	3 (17.65%)
**Need for anti-failure medications**	12 (70.59%)
**Duration of hospital stay** (days)	11 (8–15)

Abbreviations: VSD = ventricular septal defect; ICU = intensive care unit; RVOT = right ventricular outflow tract. Data are presented as mean (SD), median (IQR) or frequency (%).

## Data Availability

Unidentified data are available upon request from the corresponding author.

## Supplementary Materials

The following supporting information can be downloaded at: https://www.mdpi.com/article/10.3390/jcm15041457/s1, Video S1: Video showing the surgical technique of cylinder Bicuspid Pulmonary valve reconstruction with equine pericardium.

## Abbreviations

RVOT	Right Ventricular Outflow Tract
RVOTO	Right Ventricular Outflow Tract Obstruction
CHD	Congenital Heart Disease
BSA	Body Surface Area
CPB	Cardiopulmonary Bypass
PPS	Peripheral Pulmonary artery Stenosis
PR	Pulmonary Regurgitation
ECMO	Extracorporeal Membrane Oxygenation
CHB	Complete Heart Block
ICU	Intensive Care Unit
SD	Standard deviation
PGs	Pressure Gradients
PTFE	Polytetrafluoroethylene

## References

[1] Wang X., Bakhuis W., Veen K.M., Bogers A.J.J.C., Etnel J.R.G., van Der Ven C.C.E.M., Roos-Hesselink J.W., Andrinopoulou E.-R., Takkenberg J.J.M. (2022). Outcomes after right ventricular outflow tract reconstruction with valve substitutes: A systematic review and meta-analysis. Front. Cardiovasc. Med..

[2] Singh S.K., Faridmoayer E., Vitale N., Woodard E., Xue Y., Abramov A., Levy R.J., Ferrari G. (2025). Valved Conduits for Right Ventricular Outflow Tract Reconstruction: A Review of Current Technologies and Future Directions. Pediatr. Cardiol..

[3] Ali Y.A., Roushdy A., Hegab M.A., Mohammed A.M. (2023). Post-right ventricle to pulmonary artery conduit: Short- and intermediate-term outcomes: A single-center study. Cardiothorac. Surg..

[4] Carrel T. (2023). Past, present, and future options for right ventricular outflow tract reconstruction. Front. Surg..

[5] Taksaudom N., Thuropathum P., Tepsuwan T., Tantraworasin A., Sittiwangkul R., Phothikun A., Woragidpoonpol S. (2024). Comparison of Right Ventricular Outflow Tract Reconstruction Techniques on Mid-Term Pulmonic Valve Fate. World J. Pediatr. Congenit. Hear. Surg..

[6] Talwar S., Das A., Siddarth B., Choudhary S.K., Airan B. (2019). Patch materials for right ventricular outflow reconstruction: Past, present, and future. Indian J. Thorac. Cardiovasc. Surg..

[7] Arafat A.A., Elatafy E.E., Elshedoudy S., Zalat M., Abdallah N., Elmahrouk A. (2018). Surgical strategies protecting against right ventricular dilatation following tetralogy of Fallot repair. J. Cardiothorac. Surg..

[8] Elassal A.A., Al-Radi O.O., Zaher Z.F., Dohain A.M., Abdelmohsen G.A., Mohamed R.S., Fatani M.A., Abdelmotaleb M.E., Noaman N.A., Elmeligy M.A. (2021). Equine pericardium: A versatile alternative reconstructive material in congenital cardiac surgery. J. Cardiothorac. Surg..

[9] Kubota H., Endo H., Noma M., Tsuchiya H., Yoshimoto A., Takahashi Y., Inaba Y., Matsukura M., Sudo K. (2012). Equine pericardial roll graft replacement of infected pseudoaneurysm of the ascending aorta. J. Cardiothorac. Surg..

[10] Wu M., Fan C., Liu J., Iroegbu C.D., Chen W., Huang P., Tang M., Wu X., Wang C., Xiang K. (2022). Individualized right ventricular outflow tract reconstruction using autologous pulmonary tissue in situ for the treatment of pulmonary atresia with ventricular septum defect. Rev. Cardiovasc. Med..

[11] Iop L., Palmosi T., Sasso E.D., Gerosa G. (2018). Bioengineered tissue solutions for repair, correction and reconstruction in cardiovascular surgery. J. Thorac. Dis..

[12] Vaideeswar P., Mishra P., Nimbalkar M. (2011). Infective endocarditis of the Dacron patch—A report of 13 cases at autopsy. Cardiovasc. Pathol..

[13] Ugaki S., Rutledge J., Al Aklabi M., Ross D.B., Adatia I., Rebeyka I.M. (2015). An Increased Incidence of Conduit Endocarditis in Patients Receiving Bovine Jugular Vein Grafts Compared to Cryopreserved Homograft for Right Ventricular Outflow Reconstruction. Ann. Thorac. Surg..

[14] Bhende V.V., Sharma T.S., Krishnakumar M., Ramaswamy A.S., Bilgi K., Pathan S.R. (2023). The Utility of Invengenx® Bovine Patch for Right Ventricular Outflow Tract (RVOT) Reconstruction and Augmentation in the Surgical Management of Tetralogy of Fallot (TOF): A Contemporary Study and Review of the Literature. Cureus.

[15] Ismail M.F., Elmahrouk A.F., Arafat A.A., Hamouda T.E., Edrees A., Bogis A., Arfi A.M., Dohain A.M., Alkhattabi A., Alharbi A.W. (2020). Bovine jugular vein valved xenograft for extracardiac total cavo-pulmonary connection: The risk of thrombosis and the potential liver protection effect. J. Card. Surg..

[16] Baird C.W., Myers P.O., Piekarski B., Borisuk M., Majeed A., Emani S.M., Sanders S.P., Nathan M., del Nido P.J. (2016). Photo-oxidized bovine pericardium in congenital cardiac surgery: Single-centre experience. Interact. Cardiovasc. Thorac. Surg..

[17] Neethling W.M., Strange G., Firth L., Smit F.E. (2013). Evaluation of a tissue-engineered bovine pericardial patch in paediatric patients with congenital cardiac anomalies: Initial experience with the ADAPT-treated CardioCel(R) patch. Interact. Cardiovasc. Thorac. Surg..

[18] Dohmen P.M., da Costa F., Lopes S.V., Vilani R., Bloch O., Konertz W. (2014). Successful implantation of a decellularized equine pericardial patch into the systemic circulation. Med Sci. Monit. Basic Res..

[19] Weixler V.H.M., Kuschnerus K., Romanchenko O., Ovroutski S., Cho M.-Y., Berger F., Sigler M., Sinzobahamvya N., Photiadis J., Murin P. (2023). Midterm performance of decellularized equine pericardium in congenital heart surgery. Interdiscip. Cardiovasc. Thorac. Surg..

[20] Mashali M.H., Yousef A.A., Elmahrouk A.F., Ba-Atiyah W., Rasol M.A., Arafa M.A., Shihata M.S., Jamjoom A.A., Hamouda T.E. (2023). Reintervention after repair of tetralogy of Fallot: A one-decade single-center experience. Cardiothorac. Surg..

[21] Kim H.-W., Seo D.-M., Shin H.J., Park J.-J., Yoon T.-J. (2011). Long Term Results of Right Ventricular Outflow Tract Reconstruction with Homografts. Korean J. Thorac. Cardiovasc. Surg..

[22] Boshnakov V., Mitev I., Lazarov S., Pechilkov D., Desnous B., El Louali F., Macé L., Fouilloux V., Lenoir M. (2023). Right Ventricular Outflow Tract Reconstruction in Truncus Arteriosus: A 30-Year Two-Center Comparison between Homografts and Bovine Jugular Vein. Rev. Bras. Cir. Cardiovasc..

[23] Alamri R.M., Dohain A.M., Arafat A.A., Elmahrouk A.F., Ghunaim A.H., Elassal A.A., Jamjoom A.A., Al-Radi O.O. (2020). Surgical repair for persistent truncus arteriosus in neonates and older children. J. Cardiothorac. Surg..

[24] Elmahrouk A.F., Ismail M.F., Arafat A.A., Dohain A.M., Helal A.M., Hamouda T.E., Galal M., Edrees A.M., Al-Radi O.O., Jamjoom A.A. (2021). Outcomes of biventricular repair for shone’s complex. J. Card. Surg..

[25] Ismail M.F., Elmahrouk A.F., Arafat A.A., Hamouda T.E., Alshaikh B.A., Shihata M.S., Jamjoom A.A., Al-Radi O.O. (2020). Evolution of the Norwood operation outcomes in patients with late presentation. J. Thorac. Cardiovasc. Surg..

